# Wide variation in pre-procedural blood product transfusion practices in cirrhosis: a national multidisciplinary survey

**DOI:** 10.1097/HC9.0000000000000147

**Published:** 2023-04-26

**Authors:** Natasha Janko, Ammar Majeed, Warren Clements, Michael A. Fink, John Lubel, Mark Goodwin, Amanda Nicoll, Simone I. Strasser, Siddharth Sood, Steven Bollipo, John Bate, Kaye A Bowers, Jacob George, William Kemp, Stuart K. Roberts

**Affiliations:** 1 Department of Gastroenterology, Alfred Health, Melbourne, Victoria, Australia; 2 Central Clinical School, Monash University, Melbourne, Victoria, Australia; 3 Department of Radiology, Alfred Health, Melbourne, Victoria, Australia; 4 National Trauma Research Institute, Melbourne, Victoria, Australia; 5 Victorian Liver Transplant Unit, Austin Health, Melbourne, Australia; 6 Department of Surgery, Austin Health, The University of Melbourne, Melbourne, Australia; 7 Department of Radiology, Interventional Radiology Service, Austin Hospital, Melbourne, Australia; 8 Department of Medicine, The University of Melbourne, Melbourne, Australia; 9 Department of Gastroenterology, Eastern Health, Melbourne, Victoria, Australia; 10 AW Morrow Gastroenterology and Liver Center, Royal Prince Alfred Hospital and University of Sydney, NSW, Australia; 11 Department of Gastroenterology, Royal Melbourne Hospital, Melbourne, Australia; 12 Department of Gastroenterology, John Hunter Hospital, Newcastle, Australia; 13 School of Medicine & Public Health, University of Newcastle, Callaghan, NSW, Australia; 14 Department of Gastroenterology and Hepatology, Royal Adelaide Hospital, South Australia, Australia; 15 Department of Hepatobiliary Surgery, Alfred Health, Melbourne, Victoria, Australia; 16 Department of Surgery, Monash University, Melbourne, Victoria, Australia; 17 Storr Liver Centre, Westmead Institute for Medical Research, University of Sydney, Westmead Hospital, Westmead, NSW, 2145, Australia

## Abstract

Recent guidelines recognize the limitations of standard coagulation tests in predicting bleeding and guiding pre-procedural blood component prophylaxis in cirrhosis. It is unclear whether these recommendations are reflected in clinical practice. We performed a nationwide survey to investigate pre-procedural transfusion practices and opinions of key health care stakeholders involved in managing cirrhosis.

**Methods:**

We designed a 36-item multiple-choice questionnaire to investigate the international normalized ratio and platelet cutoffs utilized to guide pre-procedural transfusion of fresh frozen plasma and platelets in patients with cirrhosis undergoing a range of low and high-risk invasive procedures. Eighty medical colleagues from all mainland States involved in managing patients with cirrhosis were invited by email to participate.

**Results:**

Overall, 48 specialists across Australia completed the questionnaire: 21 gastroenterologists, 22 radiologists, and 5 hepatobiliary surgeons. 50% of respondents reported that their main workplace did not have written guidelines relating to pre-procedural blood component prophylaxis in patients with cirrhosis. There was marked variation in routine prophylactic transfusion practices across institutions for the different procedures and international normalized ratio and platelet cutoffs. This variation was present both within and between specialty groups and held for both low and high-risk procedures. For scenarios where the platelet count was ≤ 50 × 10^9^/L, 61% of respondents stated that prophylactic platelet transfusions would be given before low-risk and 62% before high-risk procedures at their center. For scenarios where the international normalized ratio was ≥2, 46% of respondents stated that prophylactic fresh frozen plasma would be routinely given before low-risk procedures and 74% before high-risk procedures.

**Conclusion:**

Our survey reveals significant heterogeneity of pre-procedural prophylactic transfusion practices in patients with cirrhosis and discrepancies between guidelines and clinical practice.

## Graphical abstract

**Figure:**
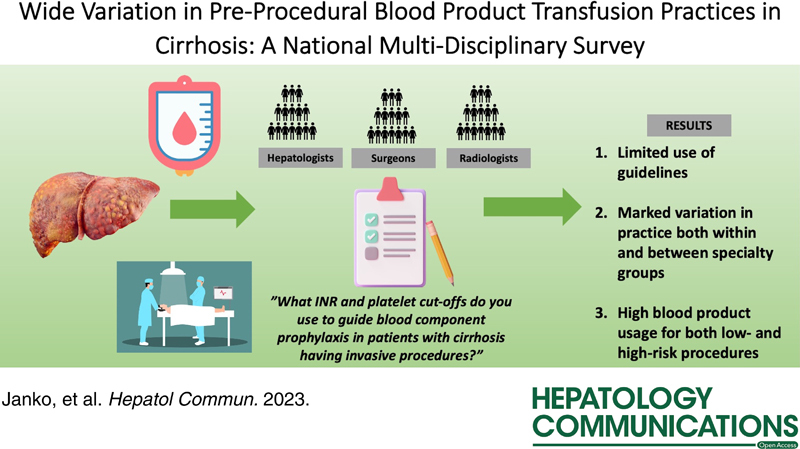

## INTRODUCTION

Patients with liver cirrhosis commonly undergo invasive procedures, both for the diagnosis and management of liver disease complications as well as their underlying comorbidities. Being able to determine a patient’s hemostatic balance is vital to the appropriate assessment of bleeding risk in the peri-procedural setting, thus forming an important component of risk management and informed consent. Standard coagulation tests, such as prothrombin time (PT)/international normalized ratio (INR) and platelet count, are frequently abnormal in patients with cirrhosis but are known to not accurately reflect bleeding risk in this setting.^1^–^6^

Over recent years, clinical practice guidelines have increasingly recognized the limitations of standard coagulation tests, particularly prothrombin time/INR, in predicting procedure-related bleeding, and as a consequence, have generally steered away from recommending pre-procedural blood component prophylaxis based on these parameters.^7^,^8^ However, given there is not as yet a validated alternative to assess hemostasis in patients with cirrhosis, there is still uncertainty regarding how best to manage cirrhotic patients in the peri-procedural setting. Although the overall risk of bleeding for patients with cirrhosis undergoing procedures remains low, particularly for low-risk procedures,^6^,^9^–^11^ there is a subset of patients who do bleed. Given this concern and the fear of litigation in some jurisdictions for failing to administer pre-procedural blood products in patients with cirrhosis, the tendency has been to attempt to “correct” abnormalities in standard coagulation tests or to mitigate any potential objective risk predictors using principles of non-maleficence. This is despite emerging evidence that there is also a risk of harm in adopting a blanket principle to administer blood products without patient-level assessment, including fluid overload and elevation in portal pressure.^12^,^13^

At present, there is a paucity of real-life data regarding prophylactic transfusion practices in patients with cirrhosis undergoing invasive procedures. This study aimed to better assess existing practice in the scope of management of peri-procedural blood results in patients with cirrhosis undergoing interventional procedures. This was addressed through a questionnaire to investigate the triggers for transfusion with fresh frozen plasma (FFP) and platelets. This was administered to clinical experts in this area across Australia, with clinicians asked to respond to the questions (1) What is the current practice at your institution? and (2) as an expert, what do you think the transfusion triggers should be? We hypothesized that the results would be heterogeneous between the different specialist groups.

## METHODS

### The questionnaire

We designed a multiple-choice online questionnaire with 3 sections, containing a total of 36 questions (see Appendix 1, http://links.lww.com/HC9/A273). The questionnaire was designed to be user-friendly and able to be completed within 5 minutes to encourage participation. Ethics approval was obtained for this project from the local human research ethics committee.

The first section included questions concerning basic information regarding the participant’s medical specialty and experience, as well as information about the institution in which they work. It also questioned respondents on whether their main workplace had available guidelines or protocols regarding the management of blood component prophylaxis in patients with cirrhosis undergoing invasive procedures, and which procedures these guidelines relate to.

The second section utilized clinical scenarios to explore the current practice at the participant’s institution regarding prophylactic blood component use in patients with cirrhosis having a range of invasive procedures. For this section, respondents were asked to answer based on what would be current practice at their institution. Scenarios were designed to include procedures with both high and low bleeding risk, in addition to a variety of platelet counts and INRs commonly encountered in patients with advanced liver disease. Each participant was asked to answer 10 clinical scenarios. The scenarios were adjusted for the speciality groups so that each participant only answered scenarios pertaining to procedures they would likely be familiar with. An example of a scenario in this section is “Cirrhotic patient planned to undergo a percutaneous liver biopsy with a platelet count of 60 × 10^9^/L and an INR of 1.6”. For each scenario, participants were given the following choices regarding what they thought would be usual practice at their workplace: Proceed without blood component prophylaxis, transfuse platelets, transfuse FFP, transfuse both platelets and FFP, or not aware of normal practice (see Appendix 1, http://links.lww.com/HC9/A273).

The third section asked the actual participants for their own individual expert opinion on what the transfusion triggers should be for prophylactic transfusion of fresh frozen plasma and platelets for patients with cirrhosis undergoing a variety of procedures. Nine different procedures/procedure types were included in this section, with participants only being asked to answer for procedures that clinicians within their specialty would usually be familiar with. If participants were unsure or did not perform a certain procedure, they were able to answer not applicable or unsure. The third section comprised 2 questions regarding whether participants currently look at additional coagulation parameters (aside from the usual INR, platelet count) to determine the need for blood component prophylaxis (see Appendix 1, http://links.lww.com/HC9/A273).

The questionnaire was completed anonymously, with the respondents not asked for any identifying details regarding themselves or their institution.

### Distribution of the questionnaire and data collection

The questionnaire was distributed by means of email to medical specialists involved in the management of patients with cirrhosis undergoing invasive procedures at hospitals across Australia. Three groups of medical specialists, namely gastroenterologists, radiologists (both interventional and diagnostic), and hepatobiliary surgeons, were invited to participate, with those that agreed clicking on a link to the questionnaire, which was completed online. Specialists were selected by the investigators based on their widely recognized expertise in their respective specialties in the management of patients with advanced liver disease. Study data were collected and managed using REDCap (Research Electronic Data Capture) hosted by Alfred Health. REDCap is a secure, web-based software platform designed to support data capture for research studies.^14^,^15^ In total, 80 specialists were invited to participate; 30 gastroenterologists and/or hepatologists, 20 hepatobiliary surgeons, and 30 radiologists.

### Definitions

Procedures were classified into low risk and high risk for bleeding based on the recently published American Gastroenterology Association guidelines.^16^ Examples of low-risk procedures included large volume abdominal paracentesis (LVAP), endoscopy with variceal banding for primary prophylaxis, and peripherally inserted central catheter insertion. Examples of high-risk procedures included liver biopsy, HCC treatments such as radiofrequency ablation and trans-arterial chemoembolization, and minor and major surgical procedures such as laparoscopic hernia repair and laparotomy, respectively.

### Data analysis

All data analysis was performed using STATA version 15.1. Descriptive data are presented as the total number or percentages of participants responding in each category.

## RESULTS

### PART 1

#### Respondents and their clinical experience

Overall, 48 specialists completed the questionnaire out of the 80 that were invited (60%): 21 of 30 gastroenterologists (70%); 22 of 30 radiologists (16 interventional radiologists and 6 diagnostic radiologists[73%]); and 5 of 20 (25%) hepatobiliary surgeons. We had representation from specialists working in 5 out of the 8 Australian states and territories: Victoria (n=24); New South Wales (n=11); Queensland (n=5); South Australia (n=4); and Western Australia (n=4). The majority of respondents (58%) worked in both private and public practice, while 40% worked purely in public institutions, and only 2% worked solely within the private sector.

Overall, the respondents had a great deal of clinical experience, with 65% having over 10 years of experience and 23% having over 20 years of experience working in their specialty area. As expected, gastroenterologists/hepatologists cared for more patients with cirrhosis per week than radiologists and hepatobiliary surgeons, with most gastroenterologists caring for more than 10 patients with cirrhosis per week, compared with radiologists and surgeons who cared for fewer than 5. Table 1 outlines the characteristics of the respondents in further detail.

**TABLE 1:** Characteristics of survey respondents

	Gastroenterologists (n=21)	Radiologists (n=22)	Surgeons (n=5)
Years worked in the speciality (n, %)			
< 5 y	1 (5)	5 (23)	0
5–9 y	4 (19)	7 (32)	0
10–14 y	4 (19)	6 (27)	3 (60)
15–20 y	6 (29)	0	1 (20)
> 20 y	6 (29)	4 (18)	1 (20)
State (n,%)			
Victoria	6 (29)	15 (68)	3 (60)
New South Wales	6 (29)	3 (14)	2 (40)
Queensland	4 (19)	1 (5)	0
South Australia	3 (14)	1 (5)	0
Western Australia	2 (10)	2 (9)	0
Northern Territory	0	0	0
Tasmania	0	0	0
Type of practice (n, %)			
Public practice only	9 (43)	10 (45)	0
Private practice only	1 (5)	0	0
Private and public practice	11 (52)	12 (55)	5 (100)
No. of patients with cirrhosis managed per week (n, %)			
<5	1 (5)	13 (59)	5 (100)
5–10	5 (24)	7 (32)	0
11–20	6 (29)	1 (5)	0
>20	9 (43)	1 (5)	0

#### Institutional guidelines or protocols on the management of blood component prophylaxis in patients with cirrhosis having invasive procedures

Sixteen specialists (33%) responded that their main workplace did have written guidelines or protocols relating to blood component prophylaxis in patients with cirrhosis undergoing invasive procedures, while 24 (50%) said there were no such guidelines available, and 8 (17%) were unsure. When asked which of the following procedures these guidelines related to, the 16 respondents with available guidelines answered as follows: liver biopsy (16, 100%), LVAP (13, 81%), endoscopy (7, 44%), central venous catheter insertion (7, 44%), minor surgery (6, 38%), and major surgery (9, 56%).

Six out of the 16 (37.5%) respondents who were aware of available guidelines thought these were based on scientific evidence. However, no one felt this evidence was strong, with 3 (50%) believing the evidence to be weak and 3 (50%) believing it to be moderate. Eight respondents (50%) considered that the guidelines were likely to be based on expert consensus, and 2 (12.5%) on individual opinion.

### PART 2: Routine practice across institutions

#### Platelet transfusion triggers for low-risk and high-risk procedures

**FIGURE 1:**
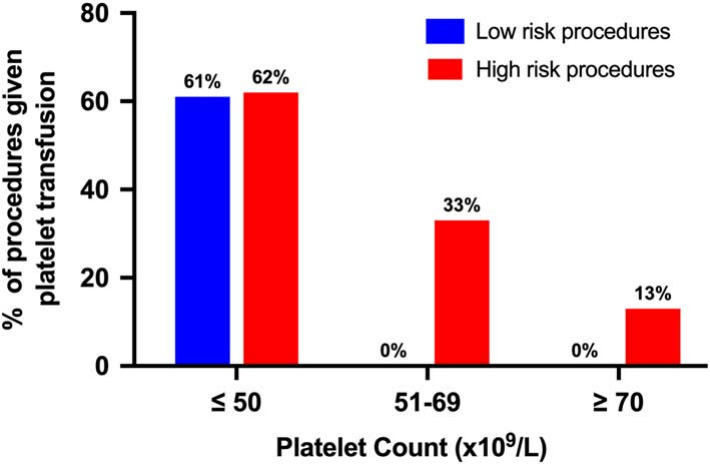
Percentage of proceduralists who give platelet transfusion according to platelet cutoff for low and high-risk procedures (Current practice).

Figure 1 details the percentage of proceduralists that currently give prophylactic platelet transfusion according to various platelet cutoffs for low-risk and high-risk procedures. Overall, in clinical scenarios where the platelet count was ≤ 50 × 10^9^/L, 61% of respondents stated that prophylactic platelet transfusions would be given before low-risk and 62% before high-risk procedures. With increasing platelet count, prophylactic platelet transfusion would be given before procedures at a smaller percentage of institutions. For scenarios where the platelet count was ≥ 70 × 10^9^/L, no participants said that prophylactic platelet transfusion would be given for a low-risk procedure, and only 13% said it would be given for a high-risk procedure.

#### INR transfusion triggers for low-risk and high-risk procedures

**FIGURE 2:**
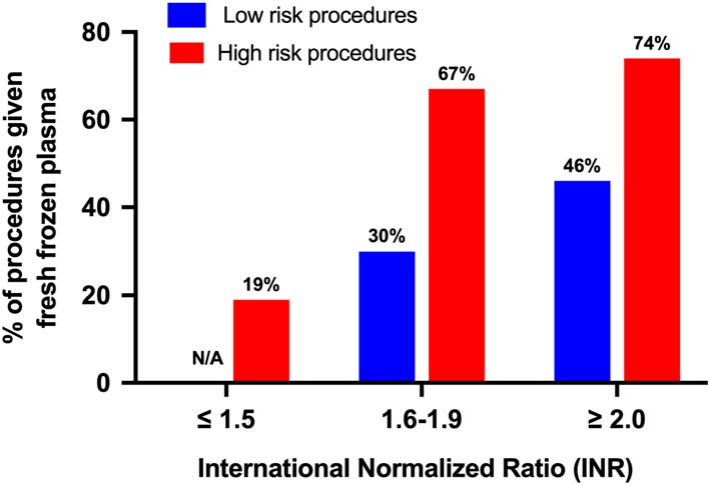
Percentage of proceduralists who give prophylactic fresh frozen plasma transfusion according to INR cutoff for low and high-risk procedures (Current practice).

Figure 2 displays the percentage of proceduralists who currently give FFP prophylaxis before low-risk and high-risk procedures according to various INR cutoffs. For scenarios where the INR > 2.0, 46% of respondents stated that prophylactic FFP would be routinely given before low-risk procedures and 74% before high-risk procedures. With increasing INR, prophylactic FFP transfusion would be given before a greater percentage of procedures. This trend was seen for both low-risk and high-risk procedures; however, for each INR range, a greater percentage of proceduralists currently give FFP prophylaxis for high-risk procedures than for low-risk procedures.

#### Variation in transfusion practices across proceduralists/institutions

There was marked variation in responses regarding routine prophylactic transfusion practices across institutions for each of the procedures and INR and platelet cutoffs. Supplementary tables 1 and 2, http://links.lww.com/HC9/A272 show the use patterns of prophylactic FFP and platelet transfusion across the different scenarios exploring low-risk and high-risk procedures, respectively. This variation was present both within and between specialist groups and held for both low-risk and high-risk procedures.

For example, 1 scenario outlined a patient undergoing an LVAP with an INR of 3.0 and platelet count of 40 × 10^9^/L. Out of the 42 participants (gastroenterologists and radiologists) who responded, 20 (48%) said both FFP and platelet prophylaxis would routinely be given, 11 (26%) and 4 (10%) participants said prophylaxis with FFP alone or platelets alone, respectively. The remaining 7 (17%), all gastroenterologists, asserted that no blood component prophylaxis would normally be given.

### PART 3: Individual expert opinion

#### Platelet transfusion triggers

Table 2 details what the expert respondents believe the platelet transfusion triggers should be for a range of procedures. Overall, there was a wide variation in the participants' responses, although the accepted platelet cutoff did appear to increase in line with an increase in the perceived bleeding risk of the procedure.

**TABLE 2:** Lowest platelets count that respondents were happy to accept without the need for blood component prophylaxis (% of respondents) (Expert opinion)

	Proceed at any platelet count	30 × 10^9^/L	50 × 10^9^/L	70 × 10^9^/L	100 × 10^9^/L	>100 × 10^9^/L
Low-risk procedures						
Diagnostic abdominal paracentesis	47	35	18	-	-	-
Variceal banding	43	17	40	-	-	-
Large volume abdominal paracentesis	5	40	55	-	-	-
High-risk procedures						
Trans-jugular liver biopsy	22.5	20	55	2.5	-	-
Transabdominal chemoembolization	2.7	13.5	70.2	10.8	2.7	-
Radiofrequency ablation	-	2.7	70.2	24.3	2.7	-
Percutaneous liver biopsy	-	2.3	60.4	32.6	4.7	-
Minor surgery*	-	-	54.1	37.5	4.2	4.2
Major surgery†	-	-	33.3	37.5	16.7	12.5

^*^ Minor surgery, for example, inguinal hernia repair.

^†^ Major surgery, for example, laparotomy.

Almost half of the expert respondents were happy to proceed at any platelet count without prophylaxis for both diagnostic abdominal paracentesis (47%) and variceal banding (43%). However, for large volume paracentesis, also a low-risk procedure, only 5% of respondents were happy for the procedure to go ahead without prophylactic platelet transfusion, with 40% accepting a minimum platelet count of 30 × 10^9^/L and a further 55% believing prophylaxis should be given to patients with a platelet count less than 50 × 10^9^/L.

With regards to high-risk procedures, a platelet cutoff of 50 × 10^9^/L was the most frequently cited transfusion threshold, with over 50% of respondents agreeing that prophylactic platelet transfusion should be given before liver biopsy, transabdominal chemoembolization, RFA and minor surgery at this count. For major surgery such as laparotomy, 33.3% thought a platelet prophylaxis cutoff of 50 × 10^9^/L was most appropriate, while 37.5% felt a higher cutoff of 70 × 10^9^/L should be utilized. No respondents felt that it was appropriate to proceed without platelet prophylaxis (regardless of platelet count) for radiofrequency ablation, percutaneous liver biopsy, or minor and major surgical procedures.

There were notable differences in responses between specialist groups. Gastroenterologists were more comfortable to proceed with LVAP at a lower platelet count compared with radiologists; the 2 specialists happy to proceed at any platelet count without prophylaxis were both gastroenterologists, and 57% of gastroenterologists were accepting of a minimum platelet count of 30 × 10^9^/L, compared with only 23% of radiologists. This is in contrast with percutaneous liver biopsy, where more radiologists (17/22, 77%) accepted a lower platelet count of 50 × 10^9^/L than gastroenterologists (9/21, 43%). More gastroenterologists (11/21, 52%) considered a higher cutoff of 70 × 10^9^/L more appropriate for percutaneous liver biopsy. Further details regarding the differences in specialist groups can be found in Supplementary Table 3, http://links.lww.com/HC9/A272.

#### INR transfusion triggers

The opinions of our experts regarding FFP prophylaxis according to specific INR cutoffs for a variety of procedures are presented in Table 3. Again, there was a wide variation in responses for each of the given procedure types.

**TABLE 3:** Highest INR that respondents were happy to accept without the need for blood component prophylaxis (% of respondents) (Expert opinion)

	1.5	1.7	2.0	2.5	3.0	Proceed at any INR
Low-risk procedures						
Diagnostic abdominal paracentesis	2.3	13.9	16.3	16.3	4.7	46.5
Variceal banding	8.7	17.4	17.4	-	8.7	47.8
Large volume abdominal paracentesis	9.3	11.6	37.2	16.2	2.3	23.2
High-risk procedures						
Trans-jugular liver biopsy	5.1	23.1	15.4	12.8	7.7	35.9
Transabdominal chemoembolization	24.3	27.0	27.0	13.5	-	8.1
Radiofrequency ablation	45.9	35.1	16.2	2.7	-	-
Percutaneous liver biopsy	65.9	24.4	4.9	4.9	-	-
Minor surgery*	60.0	24.0	12.0	4.0	-	-
Major surgery†	68.0	24.0	4.0	4.0	-	-

* Minor surgery, for example, inguinal hernia repair.

† Major surgery, for example, laparotomy.

Similar to the pattern that was seen with platelet prophylaxis, 46.5% and 47.8% of our experts were happy to proceed without FFP prophylaxis at any INR for diagnostic abdominal paracentesis and endoscopic variceal banding, respectively. The remaining half of specialists, however, believed that pre-procedural FFP should be given for varying INR cutoffs between 1.5 and 3.0 (see Table 3). For LVAP, also considered a low-risk procedure, the pattern of desired FFP prophylaxis was different, with only 23.2% of proceduralists surveyed willing to proceed without pre-procedural FFP at any INR. For LVAP, the most commonly agreed on INR cutoff for prophylactic FFP was 2.0 (37.2% of specialists).

For most high-risk procedures (percutaneous liver biopsy, minor and major surgical procedures), the majority of experts (65.9%, 60.0%, and 68.0%, respectively) agreed that an INR cutoff of 1.5 should be used to dictate the need for FFP prophylaxis. For these procedures, a minority of experts felt higher INRs between 1.7 and 2.5 could be used, but no experts were comfortable to proceed without prophylaxis at any INR. The pattern was quite different for transjugular liver biopsy, with 35.9% of specialists surveyed happy to proceed without FFP prophylaxis at any INR.

Differences in the INR transfusion trigger cutoffs advocated by the 3 specialist groups can be seen in Supplementary Table 4, http://links.lww.com/HC9/A272.

#### Consideration of other standard coagulation tests (fibrinogen level and activated partial thromboplastin time)

The majority of respondents never or rarely consider a patient’s fibrinogen (67%) or activated partial thromboplastin time (73%) when deciding on FFP prophylaxis. Of the 10 (20%) specialists who always or often consider a patient’s fibrinogen level, 7 were gastroenterologists, and 3 were radiologists. Only 8 specialists (17%) said they always or often consider a patient’s activated partial thromboplastin time; 3 gastroenterologists, 4 radiologists, and 1 surgeon.

## DISCUSSION

In our national survey of gastroenterologists, radiologists (both interventional and diagnostic), and surgeons expert in the management of patients with liver cirrhosis, this study showed significant variation in prophylactic blood component practices across institutions and for both low and high-risk procedures. Only one-third of our respondents were aware of related hospital-based guidelines, and most agreed that these were not based on high-level evidence. This study also found significant variation in specialists’ beliefs regarding what platelet count and INR transfusion threshold triggers should be. This variation was present both between and within specialist groups.

Several international organizations have recently updated their clinical practice guidelines on this topic. Both the American Gastroenterological Association Institute and the European Association for the Study of the Liver now recommend against pre-procedural laboratory hemostatic testing and routine use of blood product prophylaxis in patients with cirrhosis having invasive procedures with a low risk of bleeding.^7^,^8^ Similarly, the Society of Interventional Radiology Consensus Guidelines do not specifically recommend against testing but do caution the interpretation of traditional platelet and INR thresholds for patients with cirrhosis and suggest that alternative higher thresholds be utilized.^17^,^18^ Despite this, this study revealed that the majority of proceduralists still recommend that FFP and platelet prophylaxis be administered based on traditional INR and platelet count thresholds, respectively, before low-risk procedures. For patients with cirrhosis and thrombocytopenia with platelets < 50 × 10^9^/L, we found no difference in current transfusion practice between low-risk and high-risk procedures, with a relatively high percentage of proceduralists giving platelet transfusions before low and high-risk procedures at 61% and 62%, respectively. With regards to FFP prophylaxis, we observed a difference in accepted INR cutoffs between low and high-risk procedures. However, many proceduralists still give FFP on the basis of INR elevation to patients having low-risk procedures (30% to patients with an INR between 1.6 and 1.9 and 46% to patients with an INR ≥ 2). Given that the vast majority of procedures performed in patients with cirrhosis are low risk, the absolute impact of adherence to guidelines could substantially limit blood product use in cirrhosis.

For patients with cirrhosis having procedures with a higher risk of bleeding, societal recommendations are less well defined, but still do not support routine INR/platelet/fibrinogen blood component prophylaxis. For this group, the American Gastroenterological Association Institute supports an individualized approach whereby patients with severe coagulation abnormalities are discussed with a hematologist.^7^ In contrast, the European Association for the Study of the Liver suggests baseline laboratory testing of hemostasis may be useful in case of a bleeding event.^8^ Conversely, the Society of Interventional Radiology guidelines suggest considering a cirrhosis-specific INR threshold of 2.5, a platelet threshold of 30 × 10^9^/L, and a fibrinogen threshold of 100 mg/dL.^17^ The paucity of evidence in this space and the absence of clear and consistent guidelines between specialist groups may perpetuate the significant heterogeneity in clinical practice that we observed in our study.

There is increasing evidence that INR/PT do not predict peri-procedural bleeding in patients with cirrhosis.^1^ Patients with cirrhosis often have an elevated INR/PT, which reflects their liver disease severity, but not a hemostatic deficit (the vast majority have preserved thrombin generation).^19^ FFP administration to correct an abnormal INR/PT has minimal effect on thrombin generation,^20^ and has also been shown to markedly increase portal pressures,^13^ which may paradoxically increase the risk of portal hypertensive bleeding. Despite this, the majority of our surveyed experts were not comfortable to proceed with low-risk or high-risk procedures without FFP prophylaxis at any INR. For high-risk procedures, an INR of 1.5 was the most agreed on transfusion trigger. No proceduralists were happy to perform a percutaneous liver biopsy, radiofrequency ablation, or minor or major surgical procedures without prophylaxis in a patient with an INR above 3.0. The thresholds given are likely to represent a cautious approach, with proceduralists (radiologists and surgeons in this example) carrying the medicolegal procedural risk and likely to be most liable in the unlikely event of litigation due to perceived negligence.

The relationship between thrombocytopenia and procedure-related bleeding is less clear, with some studies suggesting an increased rate of procedure-related bleeding in patients with cirrhosis, with a platelet count less than 50 × 10^9^/L,^21^ while others show no such relationship.^3^,^4^,^22^ This is reflected in international guidelines, which vaguely acknowledge that there is weak evidence that testing platelet count may help to identify patients with increased risk of bleeding, but nevertheless suggest against correction of thrombocytopenia before low-risk procedures based on predominantly expert consensus. In patients with severe thrombocytopenia (possibly less than 50 × 10^9^/L, and particularly less than 20 × 10^9^/L) undergoing high-risk procedures, where local hemostatic measures cannot be used, the European Association for the Study of the Liver suggests that platelet transfusion or use of thrombopoietin receptor agonists may be considered on a case-by-case basis.^8^ In our study, experts agreed that a platelet count of 50 × 10^9^/L was the most appropriate transfusion target in patients having high-risk procedures. This was also higher than the Society of Interventional Radiology suggested threshold of 30 × 10^9^/L. Interestingly, for some lower-risk procedures (LVAP and transjugular liver biopsy), half of our experts still believed platelet prophylaxis should be given to patients with a platelet count less than 50 × 10^9^/L. This cutoff seems ingrained in clinical practice, which is likely to be in a predominantly non-cirrhotic adult cohort and may relate to in vivo evidence demonstrating that a platelet count of 50–60 × 10^9^/L is required for adequate thrombin generation.^23^ Still, thrombin generation testing is imperfect in this setting and does not consider the increase in von Willebrand factor and circulating activated platelets seen in cirrhosis, which may counteract the thrombocytopenia, leading to adequate hemostasis.^24^

Despite evidence suggesting that procedure-related bleeding cannot be predicted by standard coagulation tests, we found that standard tests based on non-cirrhotic patient thresholds are still commonly used in real-life clinical practice to make decisions about pre-procedural blood component prophylaxis. Indeed, very few experts are willing to ignore severe abnormalities in these tests, particularly when patients are due to undergo high-risk procedures. This highlights the need for improved tests of hemostasis and risk stratification for patients with cirrhosis. In this context, there is increasing interest in the potential role of viscoelastic point-of-care tests, such as ThromboElastoGraphy (TEG) and Rotational ThromboElastoMetry in the assessment of hemostasis and bleeding risk stratification in patients with cirrhosis undergoing invasive procedures. Two randomized controlled trials have shown that using TEG to guide prophylactic FFP and platelets in patients with cirrhosis undergoing invasive procedures resulted in decreased blood product use without affecting bleeding outcomes.^25^,^26^ While guidelines state that there is currently insufficient evidence to recommend using global hemostasis assays such as TEG and Rotational ThromboElastoMetry for risk stratification in this setting, they show great potential in this space, particularly their successful utility to guide blood product transfusion in a variety of other clinical settings.

In our survey, we found significant variation in transfusion triggers within bleeding risk categories. For instance, while almost half of the experts surveyed were happy to proceed without prophylaxis at any INR or platelet count for variceal banding, much fewer were happy to proceed without prophylaxis for patients undergoing LVAP or trans-jugular liver biopsy, both also considered low-risk procedures. This finding is in keeping with results from a study by Stine et al., where twenty attendees of a coagulation in liver disease conference were presented with 7 scenarios examining opinions on procedural bleeding risk in patients with cirrhosis.^27^ They found respondents were less likely to agree to proceed with thoracocentesis than variceal banding despite both scenarios, including the same platelet and fibrinogen counts. The variations may be attributed to differences in perceived bleeding risk within risk categories, differences in respondents’ familiarity with the procedures, and/or the ability or perceived ability to manage bleeding with local hemostasis.

We also found variations in expert opinions by medical specialty. For a percutaneous liver biopsy, for example, 82% of radiologists preferred to go ahead without prophylaxis for a platelet count of 50 × 10^9^/L or higher, while only 43% of gastroenterologists were happy with the same cutoff, with the remaining 57% having a preference for a pre-procedural platelet count of 70 × 10^9^/L or higher. This may again be due to differences in the perceived bleeding risk by the different specialties and differences in the comfort level of the proceduralists with the procedure and their ability to manage any bleeding, should it occur.

Our study is limited by its small sample size and therefore was not powered to make statistical comparisons between each of the specialist groups. Due to the anonymity of participants, we were unable to determine whether different specialist groups within the same institution have different opinions/perspectives on blood component prophylaxis. Given that the individual procedures given in the scenarios differed according to specialty, it is also difficult to tease out whether differences in perspectives on prophylaxis are due to differences in the perceived bleeding risk of the specific procedures or due to differences between specialty groups. Over the last few years, there has been increasing focus on the potential utility of fibrinogen and global hemostatic assays (rotational thromboElastoMetry and TEG) in predicting procedure-related bleeding in patients with cirrhosis. Unfortunately, our questionnaire did not survey participants regarding their use and opinions of fibrinogen testing and replacement or the use of global hemostatic assays and/or thrombopoietin agonists in the peri-procedural setting. This was due to the limited number of questions we could reasonably include in a short, five-minute survey that was crafted as such to optimize response rates. In addition, participants in this survey were not randomly selected but rather invited based on their known expertise in their respective specialty leading to the possibility of selection bias in responses.

Despite these shortcomings, we believe our study provides valuable information on real-life institutional practices and expert opinions regarding pre-procedural blood component prophylaxis in the cirrhotic population. We had a high response rate of 60%, with a good mix of private and public institutional practice, and were able to capture data from 48 specialists across 3 subspecialties and in many institutions across 5 states of Australia.

In conclusion, this study showed significant heterogeneity of pre-procedural prophylactic transfusion practices and discrepancies between guidelines and clinical practice, highlighting the need for further research in this area *a priori* to provide the necessary evidence to underpin a more uniform approach to pre-procedural blood component prophylaxis in patients with cirrhosis. In addition, this study suggests the need to enhance the participation of different specialist groups and their representative societies to determine common grounds of agreement around which to build uniformly accepted practice guidelines in this area.

## Supplementary Material

SUPPLEMENTARY MATERIAL
