# Supplementary material for: Wide variation in pre-procedural blood product transfusion practices in cirrhosis: a national multidisciplinary survey
Source: Hepatol Commun. 2023 Apr 26;7(5):e0147. doi: 10.1097/HC9.0000000000000147 (PMC10146548; doi:10.1097/HC9.0000000000000147)
Supplement: SUPPLEMENTARY MATERIAL [file hc9-7-e0147-s002.pdf]

Blood component use in cirrhotic patients undergoing invasive procedures

Please complete the survey below.

Thank you!

Demographic information

What is your speciality?
- ◯ Gastroenterology & Hepatology
- ◯ Interventional Radiology
- ◯ General radiology
- ◯ Surgery

If surgery, please detail subtype:
- ◯ Hepatobiliary
- ◯ Gastrointestinal
- ◯ General

Years worked in your speciality:
- ◯ < 5 years
- ◯ 5 - 9 years
- ◯ 10 - 14 years
- ◯ 15 - 20 years
- ◯ > 20 years

Which state do you work in?
- ◯ VIC
- ◯ NSW
- ◯ QLD
- ◯ SA
- ◯ WA
- ◯ NT
- ◯ ACT
- ◯ TAS

Type of practice:
- ◯ Private practice only
- ◯ Public practice only
- ◯ Both private and public practice

How many cirrhotic patients do you (as an individual) care for on average per week (both inpatients and outpatients)?
- ◯ < 5
- ◯ 5- 10
- ◯ 11 - 20
- ◯ > 20

Does the main institution you work in have a protocol(s) or guidelines to manage blood product and/or platelet transfusion specifically in cirrhotic subjects having invasive procedures?
- ◯ Yes
- ◯ No
- ◯ Unsure

To which procedures does this protocol(s) or guidelines apply (tick all applicable):
- ☐ Liver biopsy
- ☐ Large volume abdominal paracentesis
- ☐ Endoscopic procedures
- ☐ Minor surgery
- ☐ Major surgery
- ☐ Central line cannulation

What do you think these protocols or guidelines are based on?
- ◯ Individual opinion
- ◯ Expert opinion
- ◯ Scientific evidence

Do you think evidence in this area is ________:

- ◯ weak
- ◯ moderate
- ◯ strong

Part 1. These questions present a number of clinical scenarios.
Please answer the following questions based on what would normally be done in your institution.

Note: None of these patients are taking warfarin as a cause of elevated INR

Cirrhotic patient planned to undergo a percutaneous liver biopsy with a platelet count of 60 x 10^9/L and an INR of 1.6.

- ◯ Proceed without blood component prophylaxis
- ◯ Transfuse platelets
- ◯ Transfuse FFP
- ◯ Transfuse both FFP and platelets
- ◯ Not aware of normal practice

Cirrhotic patient planned to undergo a laparoscopic liver biopsy with a platelet count of 60 x 10^9/L and an INR of 1.6.

- ◯ Proceed without blood component prophylaxis
- ◯ Transfuse platelets
- ◯ Transfuse FFP
- ◯ Transfuse both FFP and platelets
- ◯ Not aware of normal practice

Cirrhotic patient planned to undergo a gastroscopy with variceal banding as primary prophylaxis with a platelet count of 30 x 10^9/L and an INR of 1.8.

- ◯ Proceed without blood component prophylaxis
- ◯ Transfuse platelets
- ◯ Transfuse FFP
- ◯ Transfuse both FFP and platelets
- ◯ Not aware of normal practice

Cirrhotic patient planned to undergo a large volume abdominal paracentesis as primary prophylaxis with a platelet count of 30 x 10^9/L and an INR of 1.8.

- ◯ Proceed without blood component prophylaxis
- ◯ Transfuse platelets
- ◯ Transfuse FFP
- ◯ Transfuse both FFP and platelets
- ◯ Not aware of normal practice

Cirrhotic patient planned to undergo a laparoscopic hernia repair with a platelet count of 50 x 10^9/L and an INR of 2.5.

- ◯ Proceed without blood component prophylaxis
- ◯ Transfuse platelets
- ◯ Transfuse FFP
- ◯ Transfuse both FFP and platelets
- ◯ Not aware of normal practice

Cirrhotic patient planned to undergo a transjugular liver biopsy with a platelet count of 50 x 10^9/L and an INR of 2.5.

- ◯ Proceed without blood component prophylaxis
- ◯ Transfuse platelets
- ◯ Transfuse FFP
- ◯ Transfuse both FFP and platelets
- ◯ Not aware of normal practice

Cirrhotic patient planned to undergo a laparotomy with a platelet count of 50 x 10^9/L and an INR of 1.8.

- ◯ Proceed without blood component prophylaxis
- ◯ Transfuse platelets
- ◯ Transfuse FFP
- ◯ Transfuse both FFP and platelets
- ◯ Not aware of normal practice

Cirrhotic patient planned to undergo a radiofrequency ablation (RFA) for hepatocellular carcinoma with a platelet count of 50 x 10^9/L and an INR of 1.8.

- ◯ Proceed without blood component prophylaxis
- ◯ Transfuse platelets
- ◯ Transfuse FFP
- ◯ Transfuse both FFP and platelets
- ◯ Not aware of normal practice

Cirrhotic patient planned to undergo a gastroscopy with variceal banding as primary prophylaxis with a platelet count of 120 x 10^9/L and an INR of 2.7	◯ Proceed without blood component prophylaxis<br>◯ Transfuse platelets<br>◯ Transfuse FFP<br>◯ Transfuse both FFP and platelets<br>◯ Not aware of normal practice
Cirrhotic patient planned to undergo a peripherally inserted central catheter (PICC) with a platelet count of 120 x 10^9/L and an INR of 2.7	◯ Proceed without blood component prophylaxis<br>◯ Transfuse platelets<br>◯ Transfuse FFP<br>◯ Transfuse both FFP and platelets<br>◯ Not aware of normal practice
Cirrhotic patient planned to undergo a large volume abdominal paracentesis with a platelet count of 40 x 10^9/L and an INR of 3.0.	◯ Proceed without blood component prophylaxis<br>◯ Transfuse platelets<br>◯ Transfuse FFP<br>◯ Transfuse both FFP and platelets<br>◯ Not aware of normal practice
Cirrhotic patient planned to undergo a surgical excision of 1cm benign skin lesion with a platelet count of 40 x 10^9/L and an INR of 3.0.	◯ Proceed without blood component prophylaxis<br>◯ Transfuse platelets<br>◯ Transfuse FFP<br>◯ Transfuse both FFP and platelets<br>◯ Not aware of normal practice
Cirrhotic patient planned to undergo a radiofrequency ablation (RFA) for hepatocellular carcinoma with a platelet count of 80 x 10^9/L and an INR of 2.0	◯ Proceed without blood component prophylaxis<br>◯ Transfuse platelets<br>◯ Transfuse FFP<br>◯ Transfuse both FFP and platelets<br>◯ Not aware of normal practice
Cirrhotic patient planned to undergo a laparotomy with a platelet count of 80 x 10^9/L and an INR of 2.0	◯ Proceed without blood component prophylaxis<br>◯ Transfuse platelets<br>◯ Transfuse FFP<br>◯ Transfuse both FFP and platelets<br>◯ Not aware of normal practice
Cirrhotic patient planned to undergo a trans-arterial chemo-embolization (TACE) for hepatocellular carcinoma with a platelet count of 70 x 10^9/L and an INR of 2.1	◯ Proceed without blood component prophylaxis<br>◯ Transfuse platelets<br>◯ Transfuse FFP<br>◯ Transfuse both FFP and platelets<br>◯ Not aware of normal practice
Cirrhotic patient planned to undergo a laparoscopic hernia repair with a platelet count of 70 x 10^9/L and an INR of 2.1	◯ Proceed without blood component prophylaxis<br>◯ Transfuse platelets<br>◯ Transfuse FFP<br>◯ Transfuse both FFP and platelets<br>◯ Not aware of normal practice
Cirrhotic patient planned to undergo an open cholecystectomy with a platelet count of 100 x 10^9/L and an INR of 1.4.	◯ Proceed without blood component prophylaxis<br>◯ Transfuse platelets<br>◯ Transfuse FFP<br>◯ Transfuse both FFP and platelets<br>◯ Not aware of normal practice
Cirrhotic patient planned to undergo a transjugular, intrahepatic portosystemic shunt (TIPSS) with a platelet count of 100 x 10^9/L and an INR of 1.4.	◯ Proceed without blood component prophylaxis<br>◯ Transfuse platelets<br>◯ Transfuse FFP<br>◯ Transfuse both FFP and platelets<br>◯ Not aware of normal practice

Cirrhotic patient planned to undergo a percutaneous renal biopsy with a platelet count of 70 x 10^9/L and an INR of 1.5	◯ Proceed without blood component prophylaxis<br>◯ Transfuse platelets<br>◯ Transfuse FFP<br>◯ Transfuse both FFP and platelets<br>◯ Not aware of normal practice
Cirrhotic patient planned to undergo a laparotomy with a platelet count of 70 x 10^9/L and an INR of 1.5	◯ Proceed without blood component prophylaxis<br>◯ Transfuse platelets<br>◯ Transfuse FFP<br>◯ Transfuse both FFP and platelets<br>◯ Not aware of normal practice

Part 2. Please answer the following questions based on what you personally would do or think should be done

Section A. In a cirrhotic patient, what is the lowest platelet count at which you would be happy for the following procedure to be performed without platelet prophylaxis?

	Proceed at any platelet count	30 x 10^9/L	50 x 10^9/L	70 x 10^9/L	100 x 10^9/L	>100 x 10^9/L	Unsure
Diagnostic ascitic tap	◯	◯	◯	◯	◯	◯	◯
Large volume abdominal paracentesis	◯	◯	◯	◯	◯	◯	◯
Percutaneous liver biopsy	◯	◯	◯	◯	◯	◯	◯
Trans-jugular liver biopsy	◯	◯	◯	◯	◯	◯	◯
Gastroscopy with variceal banding	◯	◯	◯	◯	◯	◯	◯
Minor surgery (e.g. inguinal hernia repair)	◯	◯	◯	◯	◯	◯	◯
Major abdominal surgery (e.g. laparotomy)	◯	◯	◯	◯	◯	◯	◯
Trans-arterial chemo-embolisation (TACE)	◯	◯	◯	◯	◯	◯	◯
Radio-frequency ablation (RFA)	◯	◯	◯	◯	◯	◯	◯

Section B. In a cirrhotic patient, what is the highest INR at which you would be happy for the following procedure to be performed without FFP prophylaxis?

Note: patient is not on warfarin

	1.5	1.7	2.0	2.5	3.0	Proceed at any INR	Unsure
Diagnostic ascitic tap	◯	◯	◯	◯	◯	◯	◯
Large volume abdominal paracentesis	◯	◯	◯	◯	◯	◯	◯

Percutaneous liver biopsy	○	○	○	○	○	○	○
Trans-jugular liver biopsy	○	○	○	○	○	○	○
Gastroscopy with variceal banding	○	○	○	○	○	○	○
Minor surgery (e.g. inguinal hernia repair)	○	○	○	○	○	○	○
Major abdominal surgery (e.g. laparotomy)	○	○	○	○	○	○	○
Trans-abdominal chemo-embolisation (TACE)	○	○	○	○	○	○	○
Radiofrequency abalation (RFA)	○	○	○	○	○	○	○

Part 3.

How often do you consider a patient's fibrinogen level when making a decision regarding pre-procedural FFP prophylaxis?

- ○ Never
- ○ Rarely
- ○ Sometimes
- ○ Often
- ○ Always

How often do you consider a patient's APTT level when making a decision regarding pre-procedural FFP prophylaxis?

- ○ Never
- ○ Rarely
- ○ Sometimes
- ○ Often
- ○ Always
