# Supplementary material for: Wide variation in pre-procedural blood product transfusion practices in cirrhosis: a national multidisciplinary survey
Source: Hepatol Commun. 2023 Apr 26;7(5):e0147. doi: 10.1097/HC9.0000000000000147 (PMC10146548; doi:10.1097/HC9.0000000000000147)
Supplement: SUPPLEMENTARY MATERIAL [file hc9-7-e0147-s001.docx]

**Supplementary Table 1. Prophylactic blood component use in patients with cirrhosis having a range of LOW RISK procedures with different INR and platelet counts**

| **Procedure type** | **International normalised ratio** | **Platelet count (x10^9^/L)** | **Fresh frozen plasma only** | **Platelets only** | **Fresh frozen plasma and platelets** | **No blood component** | **Not aware of usual practice** |
| --- | --- | --- | --- | --- | --- | --- | --- |
| **Variceal banding (n=26)**  *Gastroenterologists* (n=21)  *Surgeons* (n=5) | 1.8 | 30 | 0 | 6 (23%)  6 (29%)  0 | 4 (15%)  2 (9%)  2 (40%) | 12 (46%)  12 (57%)  0 | 4 (15%)  1 (5%)  3 (60%) |
| **Large volume abdominal paracentesis (n = 22)**  *Radiologists* (n=22) | 1.8 | 30 | 0 | 10 (45%) | 9 (41%) | 3 (14%) | 0 |
| **Variceal banding (n = 26)**  *Gastroenterologists* (n=21)  *Surgeons* (n=5) | 2.7 | 120 | 7 (27%)  5 (24%)  2 (40%) | 0  0  0 | 0  0  0 | 15 (58%)  15 (71%)  0 | 4 (15%)  1 (5%)  3 (60%) |
| **Peripherally inserted central catheter (n = 22)**  *Radiologists* (n=22) | 2.7 | 120 | 0 | 0 | 0 | 19 (87%) | 3 (13%) |
| **Large volume abdominal paracentesis(n = 43)**  *Gastroenterologists* (n=21)  *Radiologists* (n=22) | 3.0 | 40 | 11 (26%)  6 (29%)  5 (23%) | 4 (9%)  2 (9%)  2 (9%) | 20 (47%)  5 (24%)  15 (68%) | 7 (16%)  7 (33%)  0 | 1 (2%)  1 (5%)  0 |
| **Surgical excision skin lesion 1cm (n =5)**  *Surgeons* (n=5) | 3.0 | 40 | 0 | 0 | 2 (40%) | 2 (40%) | 1 (20%) |

**Supplementary Table 2. Prophylactic blood component use in patients with cirrhosis having a range of HIGH RISK procedures with different INR and platelet counts**

| **Procedure type** | **International normalised ratio** | **Platelet count (x10^9^/L)** | **Fresh frozen plasma only** | **Platelets only** | **Fresh frozen plasma and platelets** | **No blood component** | **Not aware of normal practice** |
| --- | --- | --- | --- | --- | --- | --- | --- |
| **Open cholecystectomy (n = 26)**  *Gastroenterologists* (n=21)  *Surgeons* (n=5) | 1.4 | 100 | 0 | 0 | 1 (4%)  1 (5%)  0 | 23 (88%)  18 (86%)  5 (100%) | 2 (8%)  2 (9%)  0 |
| **Transjugular intrahepatic portosystemic shunt (n = 22)** *Radiologists* (n=22) | 1.4 | 100 | 0 | 0 | 0 | 17 (77%) | 5 (23%) |
| **Percutaneous renal biopsy (n = 43**)  *Gastroenterologists* (n=21)  *Radiologists* (n=22) | 1.5 | 70 | 8 (19%)  3 (14%)  5 (23%) | 2 (5%)  1 (5%)  1 (5%) | 7 (16%)  6 (29%)  1 (5%) | 21 (49%)  7 (33%)  14 (63%) | 5 (12%)  4 (19%)  1 (5%) |
| **Laparotomy (n = 5)** *Surgeons* (n=5) | 1.5 | 70 | 0 | 1 (20%) | 0 | 4 (80%) | 0 |
| **Percutaneous liver biopsy (n=43)**  *Gastroenterologists* (n=21)  *Radiologists* (n=22) | 1.6 | 60 | 13 (30%)  7 (33%)  6 (27%) | 4 (9%)  3 (14%)  1 (5%) | 10 (23%)  7 (33%)  3 (14%) | 15 (35%)  3 (14%)  12 (54%) | 1 (2%)  1 (5%)  0 |
| **Laparoscopic liver biopsy (n=5)** *Surgeons* (n=5) | 1.6 | 60 | 0 | 0 | 1 (20%) | 3 (60%) | 1 (20%) |
| **Laparotomy (n = 26)**  *Gastroenterologists* (n=21)  *Surgeons* (n=5) | 1.8 | 50 | 3 (11%)  2 (9%)  1 (20%) | 2 (8%)  1 (5%)  1 (20%) | 20 (77%)  17 (81%)  3 (60%) | 0 | 1 (4%)  1 (5%)  0 |
| **Radiofrequency ablation (n = 22)** *Radiologists* (n=22) | 1.8 | 50 | 6 (27%) | 1 (5%) | 9 (40%) | 5 (23%) | 1 (5%) |
| **Radiofrequency ablation (n = 43)**  *Gastroenterologists* (n=21)  *Radiologists* (n=22) | 2.0 | 80 | 27 (63%)  14 (67%)  13 (59%) | 0  0  0 | 7 (16%)  2 (9%)  5 (23%) | 7 (16%)  4 (19%)  3 (14%) | 2 (5%)  1 (5%)  1 (5%) |
| **Laparotomy (n =5)** *Surgeons* (n=5) | 2.0 | 80 | 5 (100%) | 0 | 0 | 0 | 0 |
| **Transabdominal chemoembolization (n =43)**  *Gastroenterologists* (n=21)  *Radiologists* (n=22) | 2.1 | 70 | 18 (42%)  10 (48%)  8 (36%) | 0 | 4 (9%)  2 (9%)  2 (9%) | 16 (37%)  8 (38%)  8 (36%) | 5 (12%)  1 (5%)  4 (18%) |
| **Laparoscopic hernia repair (n=5)** *Surgeons* (n=5) | 2.1 | 70 | 4 (80%) | 0 | 1 (20%) | 0 | 0 |
| **Laparoscopic hernia repair (n=26)**  *Gastroenterologists* (n=21)  *Surgeons* (n=5) | 2.5 | 50 | 3 (11%)  3 (14%)  0 | 0  0  0 | 20 (77%)  16 (76%)  4 (80%) | 0  0  0 | 3 (11%)  2 (9%)  1 (20%) |
| **Transjugular liver biopsy (n=22**) *Radiologists* (n=22) | 2.5 | 50 | 5 (23%) | 0 | 2 (9%) | 11 (50%) | 4 (18%) |

**Supplementary Table 3. Platelet transfusion triggers: number of respondents in each craft group who believe platelets should be prophylactically given at particular platelet cut-offs according to specific procedures (Expert opinion)**

|  | **Proceed without prophylaxis at any platelet count** | **30 x 10^9^/L** | **50 x 10^9^/L** | **70 x 10^9^/L** | **100 x 10^9^/L** | **>100 x 10^9^/L** | **Unsure** |
| --- | --- | --- | --- | --- | --- | --- | --- |
| **Diagnostic abdominal paracentesis (n=43)**  *Gastroenterologists* (n=21)  *Radiologists* (n=22) | 20 (47%)  12 (57%)  8 (36%) | 15 (35%)  8 (38%)  7 (32%) | 8 (18%)  1 (5%)  7 (32%) | - | - | - | - |
| **Variceal banding (n=26)**  *Gastroenterologists* (n=21)  *Surgeons* (n=5) | 10 (38%)  10 (48%)  0 | 4 (15%)  4 (19%)  0 | 9 (35%)  7 (33%)  2 (40%) | - | - | - | 3 (12%)  0  3 (60%) |
| **Large volume abdominal paracentesis (n =43)**  *Gastroenterologists* (n=21)  *Radiologists* (n=22)_ | 2 (5%)  2 (10%)  0 | 17 (40%)  12 (57%)  5 (23%) | 24 (55%)  7 (33%)  17 (77%) | - | - | - | - |
| **Transjugular liver biopsy (n=43)**  *Gastroenterologists* (n=21)  *Radiologists* (n=22) | 9 (21%)  6 (28%)  3 (14%) | 8 (19%)  6 (28%)  2 (9%) | 22 (51%)  8 (38%)  14 (64%) | 1 (2%)  1 (5%)  0 | - | 1 (2%)  0  1 (5%) | 2 (5%)  0  2 (9%) |
| **Transabdominal chemoembolization (n=37)**  *Gastroenterologists* (n=21)  *Radiologists* (n=16) | 1 (3%)  0  1 (6%) | 5 (13%)  4 (19%)  1 (6%) | 26 (70%)  13 (62%)  13 (81%) | 4 (11%)  4 (19%)  0 | 1 (3%)  0  1 (6%) | - | - |
| **Radiofrequency ablation (n=37)**  *Gastroenterologists* (n=21)  *Radiologists* (n=16) | - | 1 (3%)  0  1 (6%) | 26 (70%)  14 (67%)  12 (75%) | 9 (24%)  7 (33%)  2 (13%) | 1 (3%)  0  1 (6%) | - | - |
| **Percutaneous liver biopsy (n=43)**  *Gastroenterologists* (n=21)  *Radiologists* (n=22) | - | 1 (2%)  0  1 (5%) | 26 (60%)  9 (43%)  17 (77%) | 14 (33%)  11 (52%)  3 (14%) | 2 (5%)  1 (5%)  1 (5%) | - |  |
| **Minor surgery (e.g. inguinal hernia repair) (n=26)**  *Gastroenterologists* (n=21)  *Surgeons* (n=5) | - | - | 13 (50%)  10 (48%)  3 (60%) | 9 (35%)  8 (38%)  1 (20%) | 1 (4%)  0  1 (20%) | 1 (4%)  1 (5%)  0 | 2 (7%)  2 (9%)  0 |
| **Major abdominal surgery (e.g. laparotomy)(n=26)**  *Gastroenterologists* (n=21)  *Surgeons* (n=5) | - | - | 8 (31%)  6 (28%)  2 (40%) | 9 (35%)  6 (28%)  3 (60%) | 4 (15%)  4 (19%)  0 | 3 (12%)  3 (16%)  0 | 2 (7%)  2 (9%)  0 |

**Supplementary Table 4. International normalised ratio (INR) transfusion triggers: number of respondents in each craft group who believe fresh frozen plasma should be prophylactically given at particular INR cut-offs according to specific procedures (Expert opinion)**

|  | **1.5** | **1.7** | **2.0** | **2.5** | **3.0** | **Proceed at any International normalised ratio** | **Unsure** |
| --- | --- | --- | --- | --- | --- | --- | --- |
| **Diagnostic abdominal paracentesis (n=43)**  *Gastroenterologists* (n=21)  *Radiologists* (n=22) | 1 (2%)  0  1 (5%) | 6 (14%)  2 (9%)  4 (18%) | 7 (16%)  1 (5%)  6 (27%) | 7 (16%)  4 (19%)  3 (14%) | 2 (5%)  0  2 (9%) | 20 (47%)  14 (67%)  6 (27%) | - |
| **Variceal banding (n=26)**  *Gastroenterologists* (n=21)  *Surgeons* (n=5) | 2 (7%)  1 (5%)  1 (20%) | 4 (15%)  3 (16%)  1 (20%) | 4 (15%)  4 (19%)  0 | - | 2 (7%)  2 (9%)  0 | 11 (42%)  11 (52%)  0 | 3 (12%)  0  3 (60%) |
| **Large volume abdominal paracentesis (n =43)**  *Gastroenterologists* (n=21)  *Radiologists* (n=22) | 4 (9%)  0  4 (18%) | 5 (12%)  2 (9%)  3 (14%) | 16 (37%)  5 (24%)  11 (50%) | 7 (16%)  6 (29%)  1 (5%) | 1 (2%)  1 (5%)  0 | 10 (23%)  7 (33%)  3 (14%) | - |
| **Transjugular liver biopsy (n=43)**  *Gastroenterologists* (n=21)  *Radiologists* (n=22) | 2 (5%)  1 (5%)  1 (5%) | 9 (21%)  5 (24%)  4 (18%) | 6 (14%)  4 (19%)  2 (9%) | 5 (12%)  1 (5%)  4 (18%) | 3 (7%)  0  3 (14%) | 14 (33%)  10 (48%)  4 (18%) | 4 (9%)  0  4 (18%) |
| **Transabdominal chemoembolization (n=37)**  *Gastroenterologists* (n=21)  *Radiologists* (n=16) | 9 (24%)  5 (24%)  4 (25%) | 10 (27%)  7 (33%)  3 (19%) | 10 (27%)  6 (29%)  4 (25%) | 5 (14%)  1 (5%)  4 (25%) | - | 3 (8%)  2 (9%)  1 (6%) | - |
| **Radiofrequency ablation(n=37)**  *Gastroenterologists* (n=21)  *Radiologists* (n=16) | 17 (46%)  9 (43%)  8 (50%) | 13 (35%)  8 (38%)  5 (31%) | 6 (16%)  3 (14%)  3 (19%) | 1 (3%)  1 (5%)  0 | - | - | - |
| **Percutaneous liver biopsy (n=43)**  *Gastroenterologists* (n=21)  *Radiologists* (n=22) | 27 (63%)  13 (62%)  14 (63%) | 10 (23%)  6 (29%)  4 (18%) | 4 (9%)  1 (5%)  3 (14%) | 2 (5%)  1 (5%)  1 (5%) | - | - | - |
| **Minor surgery (e.g. inguinal hernia repair) (n=26)**  *Gastroenterologists* (n=21)  *Surgeons* (n=5) | 15 (58%)  11 (52%)  4 (80%) | 6 (23%)  5 (24%)  1 (20%) | 3 (12%)  3 (14%)  0 | 1 (4%)  1 (5%)  0 | - | - | 1 (4%)  1 (5%)  0 |
| **Major abdominal surgery (e.g. laparotomy)(n=26)**  *Gastroenterologists* (n=21)  *Surgeons* (n=5) | 17 (65%)  14 (66%)  3 (60%) | 6 (23%)  4 (19%)  2 (40%) | 1 (4%)  1 (5%)  0 | 1 (4%)  1 (5%)  0 | - | - | 1 (4%)  1 (5%)  0 |
